# Functional Role of Native and Invasive Filter-Feeders, and the Effect of Parasites: Learning from Hypersaline Ecosystems

**DOI:** 10.1371/journal.pone.0161478

**Published:** 2016-08-25

**Authors:** Marta I. Sánchez, Irene Paredes, Marion Lebouvier, Andy J. Green

**Affiliations:** Department of Wetland Ecology, Estación Biológica de Doñana, EBD-CSIC, Sevilla, Spain; Ecole normale superieure de Lyon, FRANCE

## Abstract

Filter-feeding organisms are often keystone species with a major influence on the dynamics of aquatic ecosystems. Studies of filtering rates in such taxa are therefore vital in order to understand ecosystem functioning and the impact of natural and anthropogenic stressors such as parasites, climate warming and invasive species. Brine shrimps *Artemia* spp. are the dominant grazers in hypersaline systems and are a good example of such keystone taxa. Hypersaline ecosystems are relatively simplified environments compared with much more complex freshwater and marine ecosystems, making them suitable model systems to address these questions. The aim of this study was to compare feeding rates at different salinities and temperatures between clonal *A*. *parthenogenetica* (native to Eurasia and Africa) and the invasive American brine shrimp *A*. *franciscana*, which is excluding native *Artemia* from many localities. We considered how differences observed in laboratory experiments upscale at the ecosystem level across both spatial and temporal scales (as indicated by chlorophyll-*a* concentration and turbidity). In laboratory experiments, feeding rates increased at higher temperatures and salinities in both *Artemia* species and sexes, whilst *A*. *franciscana* consistently fed at higher rates. A field study of temporal dynamics revealed significantly higher concentrations of chlorophyll-*a* in sites occupied by *A*. *parthenogenetica*, supporting our experimental findings. *Artemia parthenogenetica* density and biomass were negatively correlated with chlorophyll-*a* concentration at the spatial scale. We also tested the effect of cestode parasites, which are highly prevalent in native *Artemia* but much rarer in the invasive species. The cestodes *Flamingolepis liguloides* and *Anomotaenia tringae* decreased feeding rates in native *Artemia*, whilst *Confluaria podicipina* had no significant effect. Total parasite prevalence was positively correlated with turbidity. Overall, parasites are likely to reduce feeding rates in the field, and their negative impact on host fecundity is likely to exacerbate the difference between grazing rates of native and alien *Artemia* populations at the ecosystem level. The results of this study provide evidence for the first time that the replacement of native *Artemia* by *A*. *franciscana* may have major consequences for the functioning of hypersaline ecosystems. The strong effect of parasites on feeding rate underlines the importance of taking parasites into account in order to improve our understanding of the functioning of aquatic ecosystems.

## Introduction

Filter feeders play a major role in ecosystem functioning. They are key elements in food webs, controlling primary production, phytoplankton community structure and nutrient cycling [1,2]. They often have a keystone function [3] and play a central role in trophic cascades [4]. Studies of feeding rates in filter-feeding organisms can therefore better our understanding of ecosystem functioning and the potential impact of natural and anthropogenic stressors such as parasites, biological invasions or climate warming.

Hypersaline environments are relatively simple ecosystems providing model systems for a wide range of ecological studies including food web research, biotic and abiotic interactions, etc. The brine shrimps *Artemia* spp. (Crustacea: Anostraca), the dominant macrozooplankton in hypersaline ecosystems, are non-selective filter feeders [5,6] able to control phytoplankton density [7–9] but also feeding on other microbes and detritus [9,10]. Their grazing rates are influenced by particle size [11], food concentration [6,12] and food type [6–13], but no previous information exists on the effect of biotic factors such as parasites. In coastal salt pans, *Artemia* occur along an extensive salinity gradient constituting food webs of widely different structures and complexities, including chlorophytes, proteobacteria, cyanobacteria, archaea and protists [14–16]. Through roles in microbial food webs, as primary consumers of phytoplankton and as major prey for waterbirds [17,18], *Artemia* have a central role in food web connectivity, and changes in feeding rates may strongly influence multiple ecosystem processes. In addition, *Artemia* are intermediate hosts for a rich community of avian cestode parasites [19–21] which may themselves influence the grazing rates of their hosts.

The North American brine shrimp *A*. *franciscana* has been spread widely around the world by aquaculture activities and is threatening *Artemia* biodiversity at global scale [22]. In the Mediterranean region, it has led to the extinction of many native *Artemia* (*A*. *salina* and *A*. *parthenogenetica*) populations since 1980. It outcompetes native species under most conditions in the laboratory owing partly to higher fecundity, and is rarely observed in coexistence with them in the field [23,24]. Its high competitiveness suggests *A*. *franciscana* may have a superior grazing rate compared to native *Artemia*, but this has not previously been tested. *A*. *franciscana* also benefits from enemy release in the introduced range. Whereas native populations in North America are subject to a high prevalence of avian cestodes [25], in the Mediterranean region *A*. *franciscana* is relatively resistant to native cestodes which are more prevalent in native *Artemia* and have a stronger influence on the fecundity and colour of native hosts [26,27]. Cestodes induce an increase in carotenoid, hemoglobin and lipid levels in native *Artemia* [28–31], suggesting that a cause-effect relationship with feeding rates may exist (see below). For example high levels of carotenoids observed in *Artemia* infected by some cestode parasites may be the consequence of increased feeding on carotenoid-rich algae. Parasites also induce surface and photophilic behavior, and so influence the diet [10], and even increase resistance of *Artemia* to arsenic poisoning [32].

**Fig 1:**
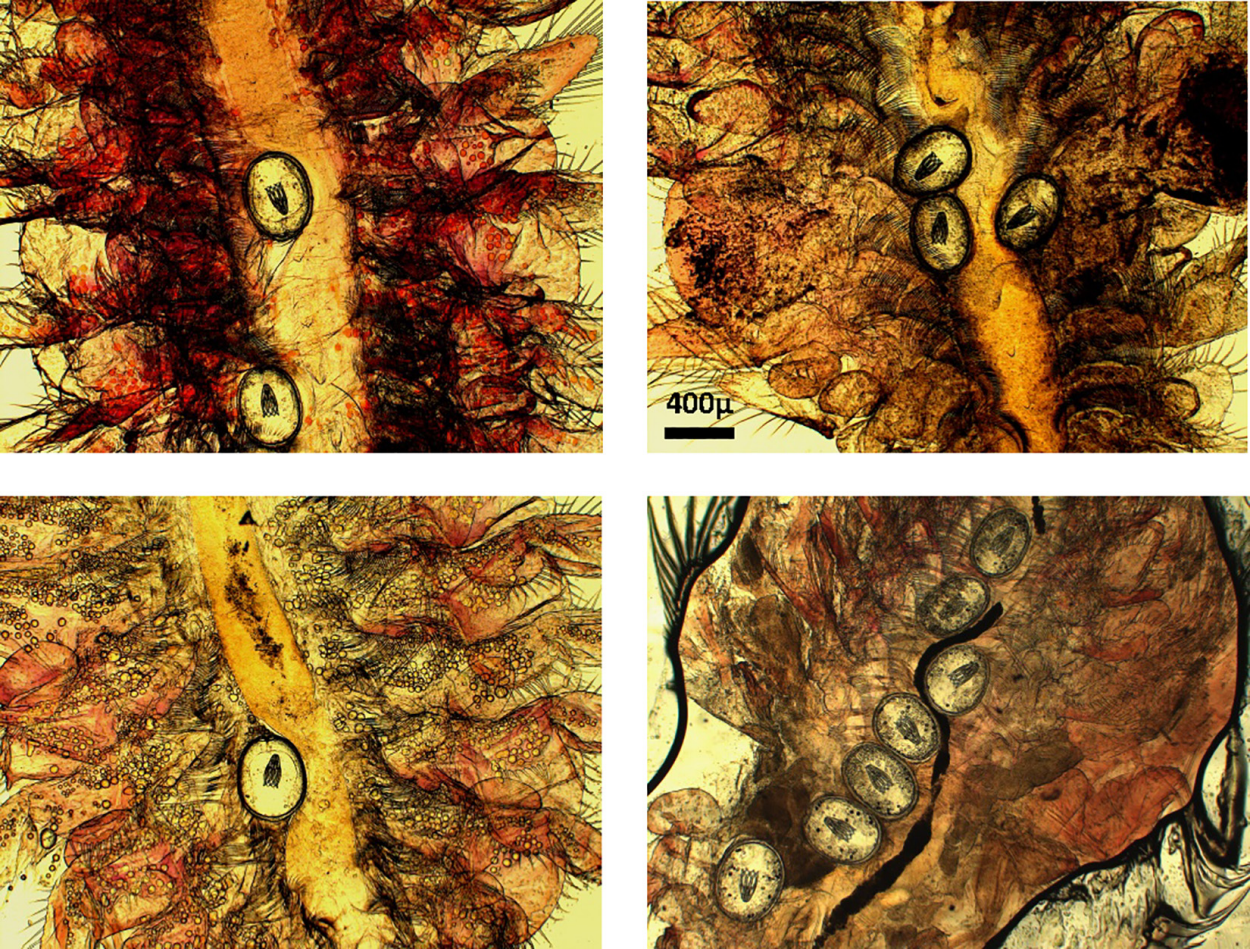
Cysticercoids of *F*. *liguloides* in the thorax of *Artemia*. Cysticercoids of *F*. *liguloides* in the thorax of *Artemia parthenogenetica*, illustrating how they can constrict the digestive tube.

Different cestode species may be expected to have contrasting effects on grazing rates as they differ markedly in size and physiological effects. *Flamingolepis liguloides* (which uses flamingos as the final host) has a particularly big cysticercoid and a preference for the host thorax (Fig 1) where it may physically interfere with feeding. Alternatively, we may expect higher feeding rates in parasitized individuals to compensate for energy being diverted to the parasite, as observed in *Gammarus pulex* infected with acanthocephalans [33]. In contrast, *Confluaria podicipina* (grebes as final host) has a very small cysticercoid but induces extensive lipid reserves in the host, suggesting it may enhance feeding rate. Cestodes may also reduce *Artemia* grazing rates at the population level by increasing avian predation rates [34] and reducing host fecundity [27] and hence population growth rates.

The main aim of this study is to compare feeding rates of the keystone filter feeders *A*. *parthenogenetica* and *A*. *franciscana*, and to test the effect of highly prevalent parasites in native species. We consider how differences observed in the laboratory upscale at the ecosystem level across both spatial and temporal scales (as indicated by chlorophyll-*a* concentration and turbidity). The specific objectives of this study are as follows:

1. To use a multifactorial design to compare the feeding rate of native and invasive *Artemia* at different temperatures and salinities under controlled laboratory conditions. These are the two most important abiotic variables affecting *Artemia*, and can have important interactions [35,36]. Our working hypothesis was that feeding efficiency would be higher in *A*. *franciscana* compared to *A*. *parthenogenetica* at all temperature and salinity combinations.
2. Using a similar method, to determine the effect of the most prevalent cestode parasites on the feeding rate of native *A*. *parthenogenetica* (cestodes were too rare in *A*. *franciscana* to enable a similar comparison).
3. Using field data, to relate the density and/or parasite prevalence of different *Artemia* taxa to temporal and spatial variation in chlorophyll-*a* concentration and turbidity. We predicted a negative correlation between *Artemia* biomass and both chlorophyll-*a* and turbidity, and that sites occupied by *A*. *franciscana* would have a lower chlorophyll-*a* concentration than those occupied by *A*. *parthenogenetica*, owing to a higher feeding rate and a reduced impact of cestodes on population abundance in the former. In this field study we controlled for salinity which can influence chlorophyll-*a* concentration, e.g. due to increased abundance of chlorophytes at higher salinities [37].

## Material and Methods

### Feeding rate experiments

#### *Artemia franciscana* vs *A. parthenogenetica*

*Artemia* sampling was conducted during November 2012 in two different salt pan complexes located along the Atlantic coast of South West Spain. E. Martínez, Director of Marismas del Odiel Natural Park, provided permission to work in the salt ponds (Junta de Andalucía 1059 Autorización DGGMN). Native *A*. *parthenogenetica* was collected from Odiel saltpans (Huelva 37°15'29"N, 6°58'25"W). Invasive *A*. *franciscana* were collected from La Tapa saltpans (Cádiz Bay 36°35’52”N, 06°13’07”W). In each locality, *Artemia* were collected from two separate evaporation ponds with distinct salinities (90 and 145 g/l). Both saltpans shared similar climatic conditions, but aquaculture projects led to the introduction of *A*. *franciscana* in Cádiz Bay [22]. *Artemia* was sampled using a 0.5 mm mesh net, then they were immediately transported to the laboratory and transferred to plastic tanks containing aerated water from the same pond, and subjected to a natural photoperiod.

Feeding rates were quantified at two salinities (90 and 145 g/l) and two temperatures (15 and 24°C). These values are representative of the range of conditions experienced by both *Artemia* populations in the field [17]. Prior to measuring feeding, individuals were acclimated for 12 hours in climatic chambers under experimental conditions (15°C-90g/l, 15°C-145g/l, 24°C-90g/l or 24°C-145g/l). Individuals were assigned to the experimental salinity that matched what they were exposed to in the field. We then selected 48 adult brine shrimps of similar size for each temperature-salinity treatment. We transferred them to Petri plates containing autoclaved and filtered (47-mm diameter glass fiber filters, 0.45 μm pore size) water from the pond at the same salinity and temperature during 1h without food, in order to increase feeding motivation.

To measure feeding rates, brine shrimps were placed individually into multi-well plates filled with 2.5 ml of the corresponding salinity treatment pond water containing 0.2 mg/ml freeze-dried green algae *Tetraselmis chuii* (EasyAlgae®, Spain) and placed in climatic chambers at 24°C and 15°C. We prepared control and blank samples in triplicate for each treatment. *Artemia* individuals grazed during 4 hours under continuous light conditions. During this period, we gently agitated the water every 30 min with a plastic Pasteur pipette, to avoid food particle sedimentation at the bottom of the plates. At the end of the experiment we collected 1ml from each well and counted remaining algal particles using an EasyCyte Plus System flow cytometer (Guava Express Plus software). The number of consumed cells was calculated by subtracting the final number of cells from the initial number. For triplicate controls and blanks, samples of 1 ml were taken and counted before and after the experiment. Brine shrimps were anaesthetized with carbonated water before being mounted (sacrificed) in a temporary glycerol mount and examined under the microscope to confirm that no cestode parasites were present. We measured the length of each individual from the end of the abdomen (furca) to the top of the head using a stereomicroscope coupled with a videocamera (Axiovision software).

#### Infected vs uninfected *A*. *parthenogenetica*

Artemia sampling was conducted at Odiel saltpans in spring 2013, from ponds of intermediate salinity where the prevalence of the cestodes Flamingolepis liguloides (hereafter FL), Anomotaenia tringae (AT, a shorebird parasite) and Confluaria podicipina (CP) was high (FL: pond E15 at 130g/l on 02/05/2013; AT and CP: pond E18 at 170g/l on 04/06/2013). We used the above experimental setup but with fixed temperature and salinity conditions. We conducted two independent experiments on the above dates, one for FL and another for CP-AT under similar conditions. Experiments were carried out at 130g/l salinity with a 0.2mg/l concentration of Tetraselmis chuii, calculating feeding rates as described above. Individuals collected from pond E18 were first acclimated to the experimental salinity for 12h. After 4 hours, all individuals were measured and their parasitic status confirmed as described above. Parasite identification followed Georgiev et al. [19]. For the first experiment we used 43 non-infected (hereafter NI) individuals and 55 infected with FL; for the second experiment we used 40 NI Artemia, 14 infected with CP and 27 with AT.

### Field study

#### Temporal variation of chlorophyll-*a* concentration in relation to *Artemia* density and species

Samples of *A*. *parthenogenetica* (from Odiel) and *A*. *franciscana* (from La Tapa) were collected monthly (from April to December 2011) from three to four ponds of different salinity, by sweeping water at each point during 15 seconds from the entire water column (15–30 cm depth) using a net of 0.1 mm mesh. Given the highly patchy distribution of *Artemia* in the field, 10 to 20 points were selected at random from different parts of the pond including the center and shoreline. In some ponds there were no *Artemia*, for reasons that are unclear but are likely to include the abundance of predators such as fish at low salinities.

At each pond, we measured temperature and salinity (with a refractometer) and collected unfiltered water samples for analysis of concentrations of chlorophyll-*a* (as a measure of phytoplankton abundance) and nutrients. Total nitrogen concentration (Total N) was measured by digestion with potassium persulfate [38]. Total phosphorus concentration (Total P) was measured by the phosphomolybdate method [39]. Chlorophyll-*a* analysis was performed by spectrophotometry using the trichromatic method [40]. Total (including all developmental stages: metanauplii, juveniles and adults) and adult *Artemia* density were determined in the laboratory.

#### Spatial variation in chlorophyll-*a* concentration and turbidity in relation to *A. parthenogenetica* density and parasite prevalence

On 23/04/2013 we sampled *A*. *parthenogenetica* (at Odiel) by filtering 20 l through a 0.5 mm mesh net, at nine different ponds covering a wide range of salinities (75–235 g/l, S1 Table, supplementary material). Samples of water (1 l) were taken for chlorophyll-*a* analysis, following the above procedure. Salinity was measured with a refractometer and turbidity with a Snell tube (a modified Secchi disc suitable for shallow waters). In the laboratory the density of *Artemia* (adult density, plus total density including metanauplii and juveniles) as well as the total biomass (dry mass after 24h at 50°C) was determined. Adult individuals (n = 100) from each pond were then randomly selected for calculation of parasite prevalence (using the above methods) so as to explore the effect of parasite infection on chlorophyll-*a* concentration and turbidity.

### Statistical analysis

Generalized linear models (GLM) were used to analyze the feeding rate from experiments, with the number of green algal particles ingested after 4h as the dependent variable. To compare feeding rate between *A*. *franciscana* and *A*. *parthenogenetica*, we used salinity, temperature and taxon (*A*. *parthenogenetica* female, *A*. *franciscana* female and *A*. *franciscana* male) as categorical variables, and body length as a continuous variable. The interactions taxon x salinity, taxon x temperature and salinity x temperature were also included. When effects were statistically significant, post hoc LSD tests were applied at a level of P < 0.05. To compare feeding rates between infected and non-infected *A*. *parthenogenetica* in the first experiment, we used parasitic status as a categorical variable with two levels (non-infected, infected with FL) and *Artemia* length as a continuous variable. Similarly, in the second experiment we used parasitic status as a categorical variable with three levels (non-infected, infected with AT, infected with CP) and *Artemia* length as a continuous variable. For analyses including three groups, post-hoc (LSD) tests were performed.

We also used GLMs to model chlorophyll-*a* concentration as a function of date, locality (categorical variables), salinity and *Artemia* density (continuous variables). We also performed independent analyses for ponds with and without *Artemia*. We predicted that i) chlorophyll-*a* concentration should not differ between La Tapa and Odiel when considering ponds without *Artemia*; and ii) chlorophyll-*a* concentration should be higher in Odiel compared with La Tapa for ponds with *Artemia*, owing to a greater feeding capacity by the *A*. *franciscana* population. The same analyses as for chlorophyll-*a* were performed for Total N and Total P to check for spatial variation in nutrient concentrations.

Owing to the small sample size (n = 9 ponds) for the study of spatial variation in chlorophyll-*a* concentration and turbidity in Odiel on 23/04/2013, we were not able to perform GLM analysis. Instead, nonparametric Spearman's rank correlation tests were performed to examine the associations between chlorophyll-*a* concentration, turbidity or salinity and *A*. *parthenogenetica* density and parasite prevalence (total prevalence, FL prevalence and CP prevalence, because prevalence of AT was very low on the date of sampling). We also correlated chlorophyll-*a* concentration with salinity.

All statistical analyses were conducted using STATISTICA software (version 12 StatSoft, Tulsa, OK).

## Results

### Feeding rate experiments

#### *A*. *franciscana* vs *A*. *parthenogenetica*

The number and body length of *Artemia* used in the feeding experiments were: 175 *A*. *parthenogenetica* (7.86 ± 0.21 mm, mean ± s.e., all females) and 360 *A*. *franciscana* (179 males: 7.32 ± 0.07, 181 females: 8.56 ± 0.10).

**Fig 2:**
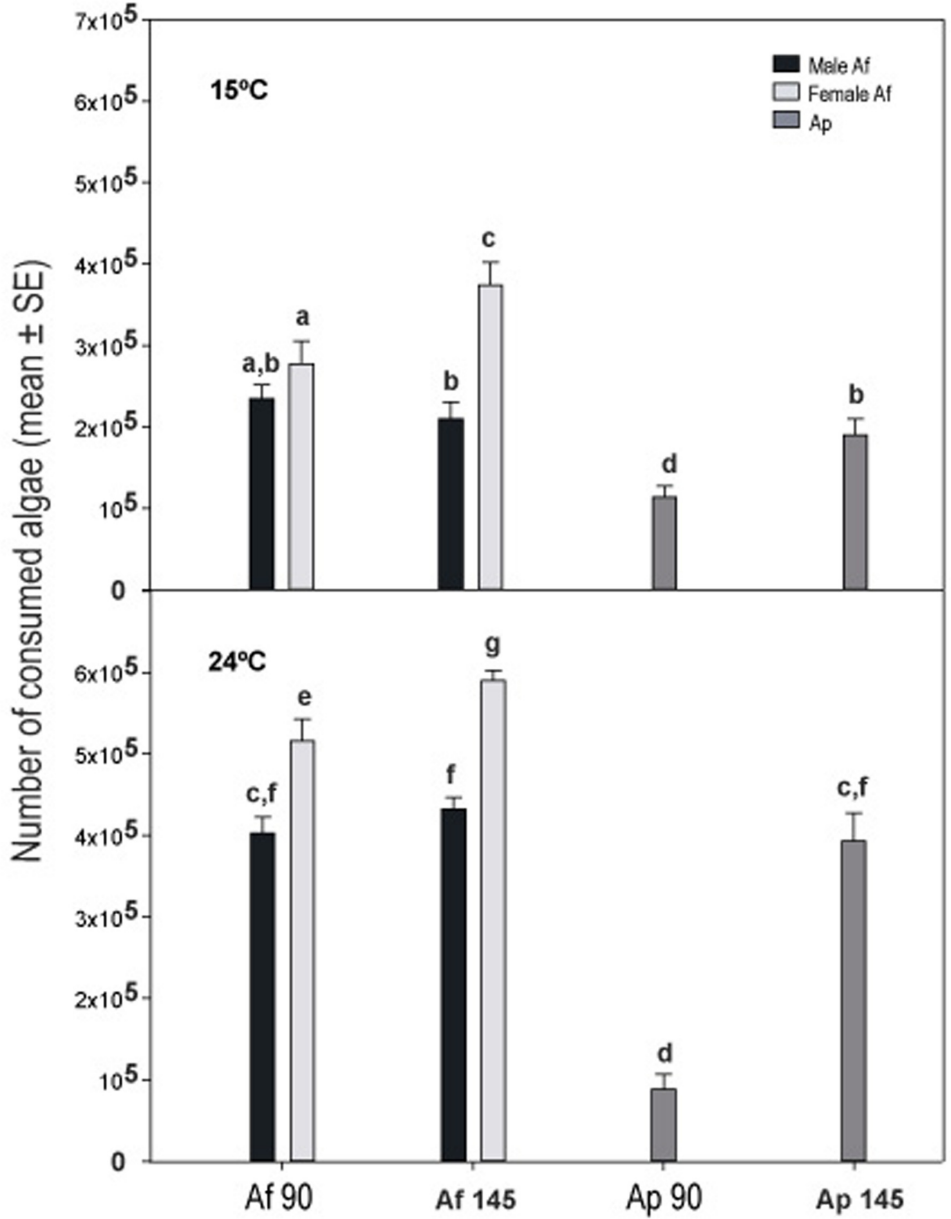
Feeding rate in *Artemia*. Feeding rate of unparasitized *A*. *parthenogenetica* and *A*. *franciscana* at different temperatures and salinities. Statistically significant differences among treatments are indicated by letters above the bars (according to post hoc LSD tests). When treatments do not share a letter it indicates they are significantly different. Exact P values are shown in S2 and S3 Tables.

**Fig 3:**
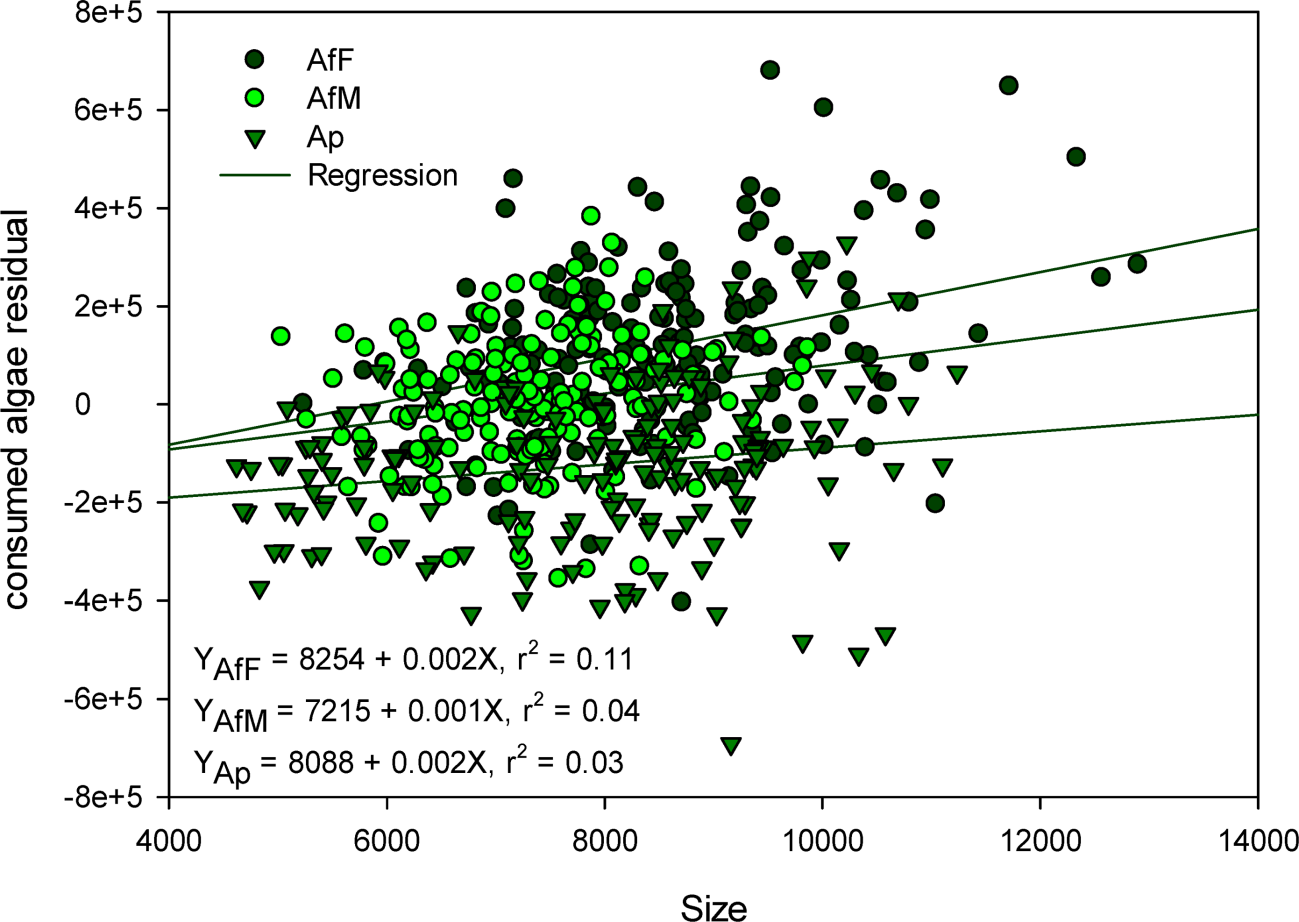
Linear regressions between rate of algae consumption and body length. Linear regressions between rate of algae consumption and body length in different *Artemia* taxa based on the experiment of Table 1. The y axis represents residuals from a GLM containing salinity and temperature as predictors, together with the interaction salinity x temperature. Ap: *A*. *parthenogenetica*, AfF: *A*. *franciscana* female, AfM: *A*. *franciscana* male.

Feeding rates generally increased with both temperature and salinity in both *Artemia* species and sexes (Fig 2). For a given combination of temperature and salinity, feeding rate was always highest in female *A*. *franciscana* and lowest in *A*. *parthenogenetica* females (Fig 2). Accordingly, GLM analysis showed highly significant effects of salinity, temperature, taxa and body length (Fig 3), with significant taxa*salinity, taxa*temperature and salinity*temperature interactions (Table 1). Larger individuals consistently had higher feeding rates (Fig 3). Almost all pairwise differences between taxa-salinity treatments and taxa-temperature treatments were significant with the exception that *A*. *franciscana* males did not change their feeding rates significantly with a change in salinity (S2 and S3 Tables).

**Table 1:** GLM on the number of cells consumed by unparasitized *Artemia*.

	Level of Effect	ESTIMATES	df	SE	F	P
Intercept		102260	1	37479.37	7.44	*0*.*0066*
Taxa	Ap	-117151	2	8372.95	112.14	*< 0*.*0001*
	AfM	16551		8778.03		
salinity g/L	90	-51839	1	5994.07	74.79	*< 0*.*0001*
temperature °C	15	-82312	1	5888.09	195.42	*< 0*.*0001*
length (μm)		28	1	4.68	35.10	*< 0*.*0001*
Taxa*salinity g/L	Ap90	-39958	2	8413.59	16.67	*< 0*.*0001*
	AfM90	43401		8307.42		
Taxa*temperature °C	Ap15	37837	2	8362.08	10.48	*< 0*.*0001*
	AfM15	-13936		8301.64		
salinity g/L*temperature °C	90g/l*15°C	16739	1	5918.13	8.00	*0*.*0049*

Results of GLM analysis on the number of cells consumed by unparasitized *Artemia* as a function of taxa (AfM = *A*. *franciscana* male, AfF = *A*. *franciscana* female, Ap = *A*. *parthenogenetica*), body length, temperature and salinity. Taxon AfF, salinity 145g/l and temperature 24°C are not included because they would be redundant (i.e. they are aliased), but they are effectively zero. Overall effects of each predictor are given with F values, with df of the denominator 527. Significant effects are shown in italics.

#### Infected vs uninfected *A*. *parthenogenetica*

**Fig 4:**
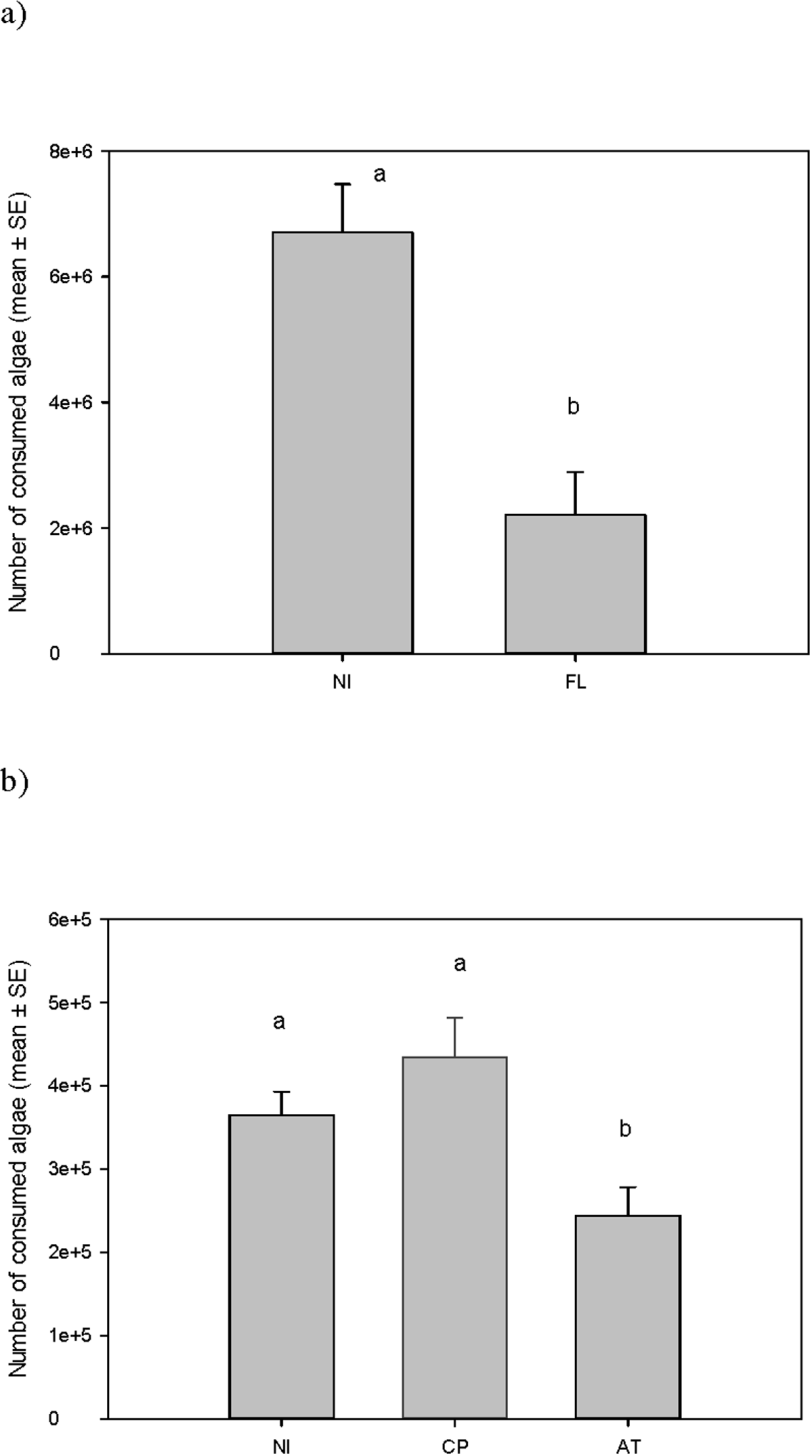
Feeding rate of infected and uninfected *Artemia parthenogenetica*. a) Feeding rate of non-infected (NI) and *F*. *liguloides*-infected (FL) *Artemia*. b) Feeding rate of non-infected *Artemia* (NI) and two categories of infected individuals (with *Confluaria podicipina* CP and *Anomotaenia tringae* AT). Statistically significant differences among treatments are indicated by letters above the bars (according to post hoc LSD tests). When treatments do not share a letter it indicates they are significantly different. Exact P values are shown in S4–S6 Tables.

The mean intensity (± SE) of infection of parasitized individuals used in the experiments was as follows: 1.89 ± 0.11 for FL, 1.00 ± 0.00 for CP and 1.00 ± 0.00 for AT. Infection by FL decreased the feeding rate significantly to less than one third compared with non-infected individuals (Fig 4A; 2207630 ± 681402 algae consumed for FL (mean ± SE) vs 6702410 ± 770638 for uninfected). As in the previous experiment, *Artemia* body length had a strong positive partial effect on the number of consumed algae (S4 Table).

A second experiment, using uninfected *Artemia* and two different categories of infected individuals (with CP and AT), showed significant differences in feeding rate among groups while controlling for length (Fig 4B; 434324 ± 47819 algae consumed for CP-infected, 243752 ± 34434 for AT-infected, 364713 ± 28290 for uninfected *Artemia*; S5 Table). Feeding rate was significantly lower in AT-infected *Artemia* than in those infected with CP or uninfected *Artemia*. Feeding rate did not differ significantly between CP-infected and uninfected individuals (S6 Table).

### Field study

#### Variation of chlorophyll-*a* and total nutrient concentrations in relation to *Artemia* species/locality, density and salinity

Salinity, chlorophyll-*a* concentration and *Artemia* density (*A*. *parthenogenetica* and *A franciscana*) from April to December 2011 for the two study sites (Odiel and Tapa respectively) are provided in S7 Table. Locality and date had significant effects on chlorophyll-*a* concentration, but the effects of salinity and *Artemia* density (whether total or adults) were not significant (Table 2).

**Table 2:** GLM on chlorophyll-*a* concentration (μg/L).

Effect	Level of effect	Estimate	df	SE	F	p
Intercept		26.46	1	8.37	9.99	*0*.*0028*
Salinity (g/l)		-0.02	1	0.05	0.17	0.6819
Density (ind/l)		-0.04	1	0.02	2.49	0.1218
Locality	Tapa	-7.18	1	3.14	5.21	*0*.*0273*
Date	April	-13.69	7	8.72	2.59	*0*.*0252*
	May	-10.90		14.62		
	June	-6.86		7.74		
	July	-14.24		7.72		
	September	-1.66		8.13		
	October	23.23		8.42		
	November	18.38		7.75		

Results of GLM analysis on chlorophyll-*a* concentration (μg/L) as a function of salinity (g/l), locality, adult *Artemia* density (ind/l) and date (month in 2011). The locality Tapa was occupied by *A*. *franciscana*. Month December and locality Odiel are aliased. The denominator for the F values is 44. Significant effects are shown in italics.

**Fig 5:**
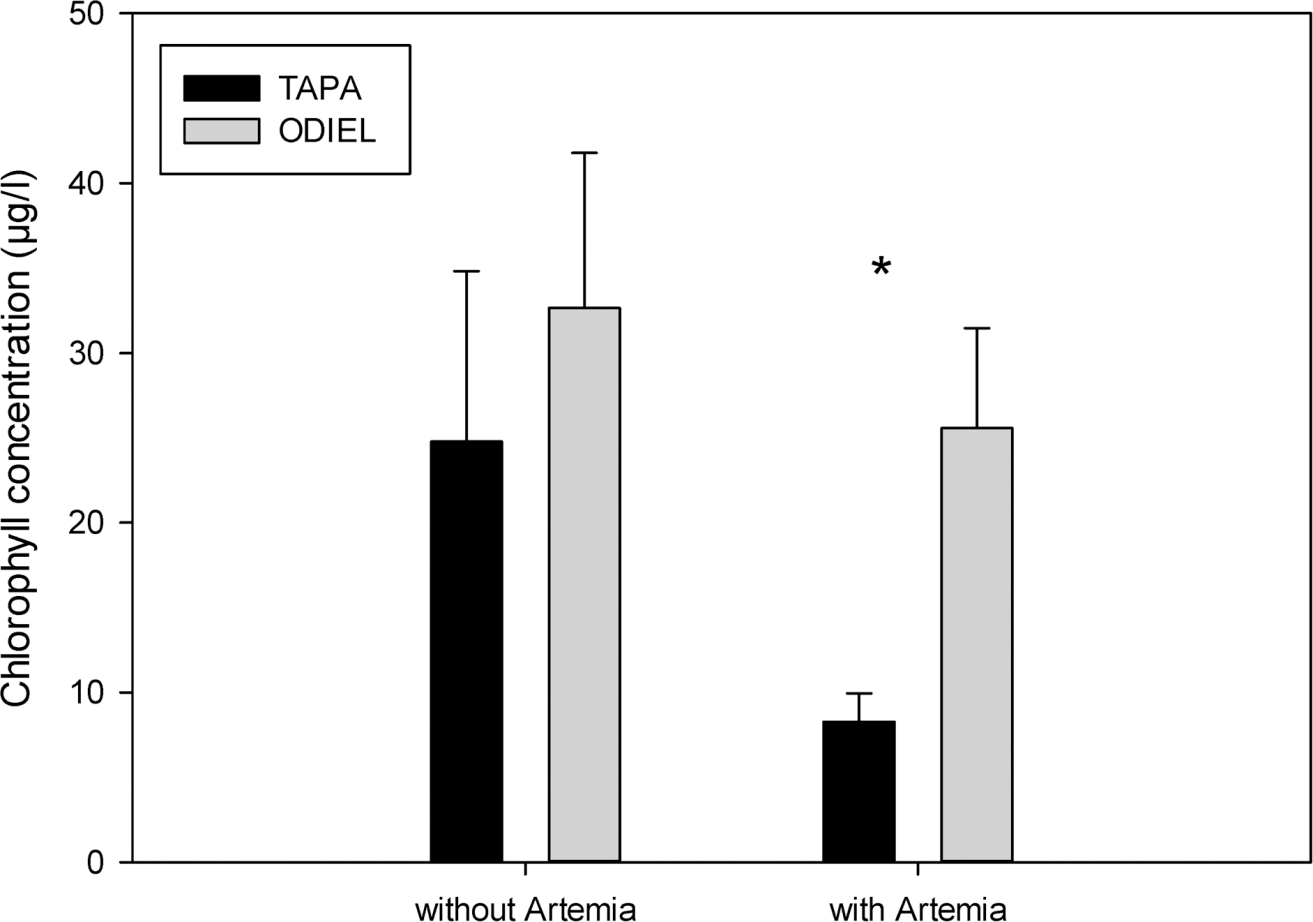
Chlorophyll-a concentration in presence and absence of *Artemia*. Comparison of chlorophyll-a concentration between la Tapa (occupied by *A*. *franciscana*) and Odiel (*A*. *parthenogenetica*) in ponds with and without *Artemia* from April to December 2011. Sample sizes: Tapa without *Artemia* = 11, Odiel without = 13, Tapa with *Artemia* = 16, Odiel with = 14. * indicates a significant difference (p = 0.006) between locations for ponds with *Artemia*.

Chlorophyll-*a* concentration was lower in La Tapa (occupied by *A*. *franciscana*). When including only ponds with *Artemia*, chlorophyll-*a* concentration remained significantly lower in La Tapa compared with Odiel (Fig 5; GLM, F_1, 20_ = 8.85, P = 0.006), although this was not the case for ponds only without *Artemia* (Fig 5; GLM, F_1, 15_ = 0.47, P = 0.53).

The GLM on Total N with locality and date as categorical variables and salinity and adult *Artemia* density as continuous variables revealed a significant positive effect of salinity on Total N (F_1, 37_ = 42.04, P < 0.0001). When considering only ponds with *Artemia*, significant effects were detected for salinity (F_1, 17_ = 29.09, P < 0.0001), date (F_7, 17_ = 5.35, P = 0.0022) and locality (F_1, 17_ = 10.17, P = 0.0053). Total N increased with salinity and was higher for Odiel. However, when including only ponds without *Artemia*, only salinity retained a significant effect (F_1, 13_ = 20.01, P = 0.00062).

Analysis of Total P showed significant effects of salinity (F_1, 27_ = 4.26, P = 0.04) and locality (F_1, 27_ = 20.29, P = 0.0001), with higher Total P at higher salinities and in Odiel. When considering only ponds with *Artemia*, significant effects were observed for date (F_6, 12_ = 3.15, P = 0.04) and locality (F_1, 12_ = 28.04, P = 0.00019). However, when considering only ponds without *Artemia*, no significant effects were detected (P > 0.07 in all the cases).

#### Spatial variation of chlorophyll-*a* concentration and turbidity in relation to *A*. *parthenogenetica* density and parasite prevalence

**Fig 6:**
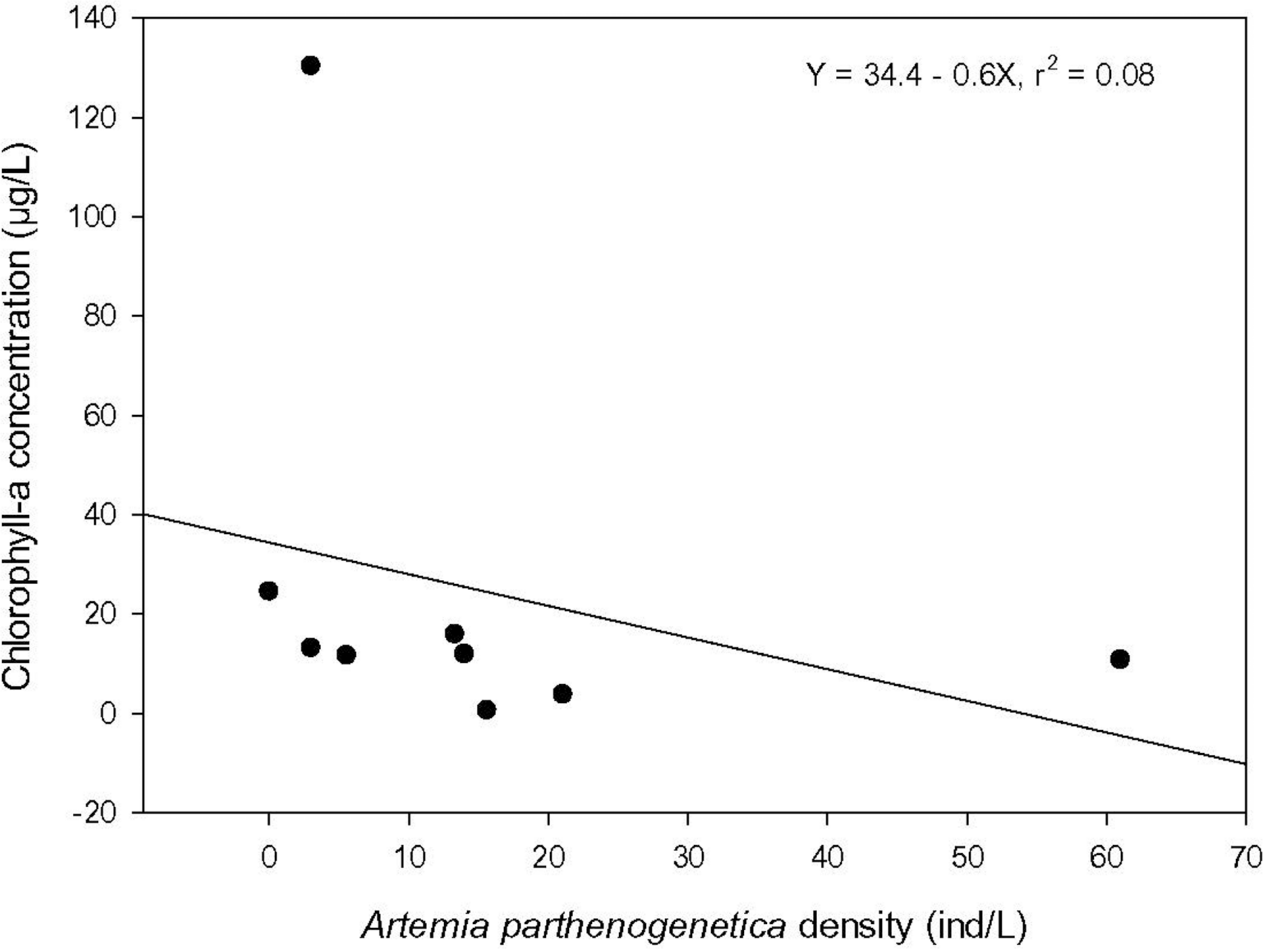
Relation between chlorophyll-a concentration and *Artemia* density. Relation between chlorophyll-a concentration and *Artemia parthenogenetica* density at Odiel saltpans measured in nine study ponds on 23/04/13.

Salinity, turbidity, chlorophyll-*a* concentration, *A*. *parthenogenetica* density and biomass, and parasite prevalence were measured in nine study ponds on 23/04/13 (S7 Table). There was no significant correlation between turbidity and adult or total *Artemia* density (r_s_ = 0.142, P = 0.676; r_s_ = -0.0251, P = 0.913, respectively) or *Artemia* biomass (r_s_ = 0.000, P = 0.983). However there was a significant negative correlation between chlorophyll-*a* concentration and total *Artemia* density (rs = -0.812, *P* = 0.00393; Fig 6), adult *Artemia* density (rs = -0.845, *P* = 0.000392) and *Artemia* biomass (rs = -0.733, *P* = 0.02). Chlorophyll-*a* concentration and turbidity were not correlated (rs = -0.500, P = 0.153, n = 9). There were no significant correlations between salinity and any of the following: *Artemia* biomass or density (p > 0.87), turbidity (P > 0.87) or Chl-*a* (P = 0.38). Neither were there significant correlations between prevalence of individual parasite species (AF and CP) and chlorophyll-*a* concentration, turbidity or salinity (p > 0.141 in all cases). Total parasite prevalence was not correlated with chlorophyll-*a* concentration or salinity (p > 0.84). However, total parasite prevalence was positively correlated with turbidity (rs = 0.683, P = 0.047, n = 8).

## Discussion

Although several studies have focused on the impacts of alien filter feeders on species diversity and community composition, very few have explored the functional consequences of the replacement of native species by invasive congeners, and the role of their parasites. Here we studied hypersaline ecosystems (and their dominant and conspicuous inhabitant, *Artemia*), which provide model systems to address these questions.

### Comparison of feeding rate in native and invasive *Artemia* and its ecological consequences

Invasive species often have a major effect on native food webs [41], and their impacts are often mediated by their feeding behaviour [42,43]. The invasion of *A*. *franciscana* is causing a dramatic loss of biodiversity in hypersaline ecosystems in several continents through the loss of native brine shrimp species and genetic diversity [23,44]. Our study indicates that *A*. *franciscana* is highly invasive partly because of faster filter-feeding (Fig 2), and that this is likely to have top-down effects on the whole food web.

Several previous studies help to explain the unusual invasiveness of *A*. *franciscana*. In laboratory studies, this species showed higher reproductive performance [23], wider eurythermal and euryhaline ranges [36] and was more resistant to pesticides [45] (but see also [46]). Field studies consistently show lower parasite loads in *A*. *franciscana* as predicted by the enemy release hypothesis [26,27,47]. This hypothesis states that species introduced in a new region should experience a decrease in regulation by natural enemies (parasites, predators, competitors), resulting in an increase in distribution and abundance [48]. In this study, we have shown that *A*. *franciscana* is also a more efficient filter feeder than native *A*. *parthenogenetica* across a range of salinities and temperatures (Fig 2). Given that *Artemia* growth, reproduction rates and brood size depend on food supply [9,49,50], our results help to explain why *A*. *franciscana* is more fecund and has higher population growth rates in the laboratory. Food limitation is likely to seriously affect other *Artemia* species in the presence of this alien congener.

To our knowledge, this represents the first field study of the ecological consequences of the *A*. *franciscana* invasion. While invasive and native *Artemia* share similar functional roles as the dominant grazers in salt ponds, differences in grazing rates may cause strong changes in the functioning of hypersaline ecosystems. Our results show lower chlorophyll-*a* concentrations in ponds with presence of *A*. *franciscana* (Fig 5), as predicted based on the results of our feeding experiments, providing the first empirical evidence that *A*. *franciscana* produces a strong top-down impact in invaded ecosystems. However, we found evidence that total nutrient concentrations were also lower in the La Tapa locality frequented by *A*. *franciscana*, providing an alternative explanation for the lower chlorophyll-*a* concentrations.

Higher feeding rates in sites invaded by *A*. *franciscana* and a concomitant decrease in density of phytoplankton such as *Tetraselmis* and *Dunaliella* can have major ramifications. For example, the increase of water clarity can be expected to favour benthic primary producers, mainly dominated by cyanobacteria. Top down effects of *Artemia* on phytoplankton are supported by the spatial correlation we recorded in the field between chlorophyll-*a* concentration and *Artemia* density and biomass (see below; Fig 6). Higher grazing rates by *A*. *franciscana* are likely to have other consequences on the food web, requiring further study. For example, a change in the rate of filter-feeding on bacteria (or indirect effects on their abundance through modified feeding rates on their competitors) will affect N dynamics and bacterial community structure [14,51]. These top-down effects can be expected to be strongest in female *A*. *franciscana*, which consumed algae at higher rates than males, perhaps to compensate for their higher reproductive investment [43]. Given the strong influence of body size on grazing rates (Fig 3), this sexual difference should be even more marked in natural conditions where females can be much bigger than males [27,52,53]. Given that *Artemia* are able to bioaccumulate pollutants [54], higher grazing rates by the exotic species could also affect the flux of contaminants through the ecosystem.

### Spatial and temporal variation in chlorophyll-*a* in relation to *Artemia* density

We found a strong negative correlation between *A*. *parthenogenetica* biomass/density and chlorophyll-*a* concentration at the spatial scale in April 2013 (Fig 6). Similar results have been previously shown for different *Artemia* populations around the world [9,55–57]. However, in 2011 the negative correlation within each locality between *Artemia* and phytoplankton abundance was not significant (P = 0.12). It is possible that because of the highly patchy spatial distribution of *Artemia* in the field, our measures of *Artemia* density were not precise enough, or that density was sometimes too low to affect phytoplankton density. Wurtsbaugh and Berry [58] showed that, at low density, *Artemia franciscana* was not able to affect chlorophyll-*a* levels in Great Salt Lake. In the case of *A*. *parthenogenetica*, cestode parasites may often keep *Artemia* at a low density (see below). Another possible reason for the weak patterns we found is that there may be time lags which our sampling regime could not detect, in which phytoplankton abundance at time *t* depends largely on *Artemia* abundance at time *t—*1. Additionally, the high proportion of metanauplii and juveniles registered throughout the year (71% on average) may contribute to our results. Reeve [6] showed that feeding rate increased with the age or size of *A*. *franciscana* metanauplii, with a difference of almost three orders of magnitude when comparing 2 and 7 day-old metanauplii. The relationship we found between body length and grazing rates (Fig 3) is consistent with this earlier work.

### The effect of parasites on feeding rate in native *Artemia* and implications for the functional role of the host

In this study we compared the effect of cestode parasites on feeding rate in native *Artemia*, but it was not possible to do the same in the invasive host due to extremely low prevalence. Many parasites alter host phenotype as part of their transmission strategy (according to the “parasite manipulation hypothesis”; [59,60]) or as a side effect of the infection. Theoretical studies suggest that parasites can have a key role in ecosystem functioning [61,62], but few empirical studies have addressed the broader ecological effects of parasite-induced phenotypic changes. Our study is one of the first to show that parasites can affect feeding rate in an invertebrate host and to demonstrate the implications for the functional role of the host.

We showed that the cestode *F*. *liguloides* (FL), the most prevalent species present in the study organisms, strongly decreased feeding rate in *A*. *parthenogenetica* (Fig 4A). Hence FL provides a further competitive advantage to the naturally more efficient filter feeder *A*. *franciscana*, because FL is far more prevalent in *A*. *parthenogenetica* than in the alien shrimp [47,63]. Similar results have been reported for acanthocephalans infecting detritivores [64]. The most plausible explanation for our results is that the big cysticercoids of FL, normally present in high numbers in the thorax, physically interfere with the feeding process or constrict the gut of the host (Fig 1). However *A*. *tringae* (AT), with a small cysticercoid, also decreased feeding rate (Fig 4B), so mechanisms other than physical interference may explain our results. For example, it may be an inevitable pathological side effect of the infection [65]. Feeding intake depression and decreased efficiency of nutrient utilisation is considered the main pathogenic effect of gastrointestinal parasites [66]. Alternatively it could be an adaptive response of the host in order to eliminate the parasite [67]. Whatever the underlying mechanism, the reduction in feeding rate by infected *Artemia* could affect different levels of ecological organisation. It is likely to have direct consequences for the host by decreasing respiration, movement, growth and energy storage. At a population level, lower feeding rates of infected individuals may benefit uninfected individuals (i.e. the ones with greatest potential for reproduction due to the effect of parasites on fecundity, [27]) by reducing competition for food.

We can also expect effects at lower trophic levels, since reduced grazing by infected *Artemia* will tend to increase phytoplankton density, and the higher cestode infection rates in native *Artemia* may contribute to the higher chlorophyll-*a* levels we recorded in Odiel (Fig 5). Although in our field study we did not detect a correlation between parasite prevalence and chlorophyll-*a* concentration, there was a positive correlation between total parasite prevalence and turbidity. *Artemia* are generalist grazers and consume a variety of protists and bacterioplankton, so by decreasing feeding rate, cestodes may reduce the ingestion of particulate organic matter (POM, see also Sánchez *et al*. [10]) which contribute to turbidity.

If we extrapolate our laboratory data on feeding rates using seasonal data of *Artemia* density and values of FL prevalence from Sánchez *et al*. [21], we can estimate that parasites can reduce grazing rates by the *Artemia* population by up to 29% (in June, when FL prevalence was 43%). This reduction may be even stronger since FL prevalence often locally reaches ≥90% (Sánchez *et al*. unpublished data). Thus, at the community level the presence of these cestode parasites may alter interactions among microbial organisms in salt ponds, in a similar way to a switch between native and alien *Artemia* (see above for discussion), as well as altering the flux of energy and nutrients through the hypersaline food web. However, our laboratory experiments are too simplistic to predict these effects in detail, especially since FL and other cestodes also change the vertical distribution of *A*. *parthenogenetica* in the water column and therefore their diet, as indicated by stable isotopes [10]. Indeed, it is possible that infection by FL has different effects on the ingestion per capita of chlorophyll-*a* and POM in the field, since infection increases the time spent near the water surface where phytoplankton are likely to be concentrated and decreases the time spent at the bottom of the water column were POM may reach higher concentrations. Furthermore, it has been shown that parasites increase longevity of infected *Artemia* allowing them to reach a larger maximum body length [28], which itself will tend to increase grazing rates. Cestode parasites castrate their hosts [27] and the reallocation of energy from reproduction to longevity/growth increases the probability of predation by and successful transmission to the avian final hosts.

Despite these limitations when attempting to extrapolate our laboratory experiment to the field situation, our results provide novel insights into the importance of parasites in the ecosystem processes, for which existing information is very limited [62]. Owing to its resistance to infection, all the above parasite-mediated ecological processes are expected to be altered by the invasion of *A*. *franciscana*, strongly affecting ecosystem functioning. Our results therefore highlight the strong ecological impact invasive species can have in ecosystems though the removal of parasites from the system.

### Conclusions

We conducted a laboratory and field study to examine feeding rates in native and invasive *Artemia* and the effect of cestode parasites, in an effort to better understand the impact of *A*. *franciscana* invasion and the functional role of different *Artemia* taxa in hypersaline ecosystems. *A*. *franciscana* is a more efficient filter feeder than its native congener *A*. *parthenogenetica*. Cestode parasites, which are highly prevalent in native but not the invasive species, significantly decrease feeding rate and increase turbidity. We provide the first evidence that the invasion of *A*. *franciscana* can have a strong impact in hypersaline ecosystems by decreasing plankton density, with potential for strong direct and indirect effects on microbial communities, altering the functional role of *Artemia* in the ecosystem. We found evidence that cestode parasites amplify the effects that a switch from native to alien *Artemia* has on grazing rates. Our results suggest that hypersaline ecosystems are more complex than previously acknowledged, with neglected organisms such as parasites playing a significant ecological role and increasing the complexity of interactions among more visible, free living organisms.

More extensive laboratory studies are required in the future, which compare the functional response of alien, native and parasitized *Artemia* across a full range of prey densities and for a variety of prey types [68]. Together with analysis of diet under natural conditions, this would allow a more complete picture of the impact of the invasion by *A*. *franciscana* on microbial communities in hypersaline ecosystems. Furthermore, detailed field studies using remote sensing should be used to study the influence of different *Artemia* taxa on the variety of pigments found in different planktonic or benthic taxa.

## Supporting Information

S1 Table Physico-chemical variables, *Artemia* density and parasite prevalence at Odiel. Physico-chemical variables, *Artemia* density and parasite prevalence recorded in individual salt ponds at Odiel on 23 April 2013. The column *Artemia* density includes adults, metanauplii and juveniles.
(DOCX)

S2 Table Post hoc tests for analysis from Table 1 under different salinity treatments. Post hoc tests for the differences between taxa under different salinity treatments in the GLM of Table 1. See Table 1 for more details. Significant differences are shown in italics.
(DOCX)

S3 Table Post hoc tests for analysis from Table 1 under different temperature treatments. Post hoc tests for the differences between taxa under different temperature treatments in the GLM of Table 1. See Table 1 for more details. Significant differences are shown in italics.
(DOCX)

S4 Table GLM on the number of cells consumed by *A*. *parthenogenetica* as a function of parasitic status (*F*. *liguloides* or uninfected). Results of GLM analysis on the number of cells consumed by *A*. *parthenogenetica* as a function of parasitic status (infected with *F*. *liguloides* FL or uninfected) at a salinity of 130 g/l. Parasitized status is aliased. Significant effects are shown in italics.
(DOCX)

S5 Table GLM on the number of cells consumed by *A*. *parthenogenetica* as a function of parasitic status (*C*. *podicipina*, *A*. *tringae* or uninfected). Results of GLM analysis on the number of cells consumed by *A*. *parthenogenetica* as a function of parasitic status (infected with *C*. *podicipina* CP, *A*. *tringae* AT or uninfected) at a salinity of 130 g/l. Unparasitized status is aliased. Significant effects are shown in italics.
(DOCX)

S6 Table Post hoc tests for analysis from S5 Table. Post hoc tests for the differences between parasitic status (*Confluaria podicipina* CP, *Anomotaenia tringae* AT, non-infected NI) in the GLM of S4 Table. Significant differences are shown in italics.
(DOCX)

S7 Table Salinity, chlorophyll-a concentration and *Artemia* density at Odiel and Tapa. Mean values (± SE) of salinity, chlorophyll-a concentration and *Artemia* density (*A*. *franciscana* and *A*. *parthenogenetica*) from April to December 2011 at La Tapa (invaded by *A*. *franciscana*) and Odiel (occupied by the native *A*. *parthenogenetica*).
(DOCX)
