# Supplementary material for: Functional Role of Native and Invasive Filter-Feeders, and the Effect of Parasites: Learning from Hypersaline Ecosystems
Source: PLoS One. 2016 Aug 25;11(8):e0161478. doi: 10.1371/journal.pone.0161478 (PMC4999065; doi:10.1371/journal.pone.0161478)
Supplement: S1 Table — Physico-chemical variables, Artemia density and parasite prevalence recorded in individual salt ponds at Odiel on 23 April 2013. The column Artemia density includes adults, metanauplii and juveniles. (DOCX) [file pone.0161478.s001.docx]

**S1 Table. Physico-chemical variables, *Artemia* density and parasite prevalence at Odiel.**

| **Pond** | **Salinity** | **turbidity** | **Chlorophyll** | **Artemia** | **Adult Artemia** | **Artemia** | **Total** | **Prev.** | **Prev.** |
| --- | --- | --- | --- | --- | --- | --- | --- | --- | --- |
|  | **(g/l)** | **(cm)** | **(µg/L)** | **dens (ind/L)** | **dens (ind/L)** | **dry weight (g/L)** | **Prev%** | **FL%** | **CP%** |
| E4 | 75 | 19 | 24.5056 | 0 | 0 | 0 |  |  |  |
| E12 | 130 | 17 | 3.7424 | 21.05 | 12.9 | 0.01884 | 19 | 16 | 5 |
| E13 | 140 | 10 | 130.3085 | 3 | 1 | 0.00295 | 8 | 4 | 7 |
| E15 | 140 | 15 | 10.727 | 61 | 26 | 0.06593 | 9 | 7 | 4 |
| E16 | 140 | 11.5 | 15.8711 | 13.3 | 0.8 | 0.00874 | 19 | 16 | 1 |
| E17 | 115 | 19.5 | 11.9157 | 14 | 4 | 0.01304 | 22 | 22 | 0 |
| E18 | 111 | 24 | 11.6402 | 5.55 | 2.7 | 0.004735 | 21 | 16 | 14 |
| S1 | 160 | 34 | 0.5767 | 15.6 | 8.95 | 0.012345 | 19 | 12 | 8 |
| A | 235 | 30 | 13.111 | 3 | 1 | 0.006655 | 41 | 0 | 41 |
