# Supplementary material for: Functional Role of Native and Invasive Filter-Feeders, and the Effect of Parasites: Learning from Hypersaline Ecosystems
Source: PLoS One. 2016 Aug 25;11(8):e0161478. doi: 10.1371/journal.pone.0161478 (PMC4999065; doi:10.1371/journal.pone.0161478)
Supplement: S2 Table — Post hoc tests for the differences between taxa under different salinity treatments in the GLM of Table 1. See Table 1 for more details. Significant differences are shown in italics. (DOCX) [file pone.0161478.s002.docx]

**S2 Table**. **Post hoc tests for analysis from Table 1 under different salinity treatments.**

| \| Taxa \| \| --- \| | \| salinity \| \| --- \| | \| Ap90 \| \| --- \| | \| Ap145 \| \| --- \| | \| AfM90 \| \| --- \| | \| AfM145 \| \| --- \| | \| AfF90 \| \| --- \| | \| AfF145 \| \| --- \| |
| --- | --- | --- | --- | --- | --- | --- | --- | --- | --- | --- | --- | --- | --- | --- | --- |
| Ap | 90 |  | *< 0.0001* | *< 0.0001* | *< 0.0001* | *< 0.0001* | *< 0.0001* |
| Ap | 145 | *< 0.0001* |  | 0.1346 | *0.0486* | *< 0.0001* | *< 0.0001* |
| AfM | 90 | *< 0.0001* | 0.1346 |  | 0.5946 | *0.0001* | *< 0.0001* |
| AfM | 145 | *< 0.0001* | *0.0486* | 0.5946 |  | *0.0013* | *< 0.0001* |
| AfF | 90 | *< 0.0001* | *< 0.0001* | *0.0001* | *0.0013* |  | *< 0.0001* |
| AfF | 145 | *< 0.0001* | *< 0.0001* | *< 0.0001* | *< 0.0001* | *< 0.0001* |  |
