# Supplementary material for: Functional Role of Native and Invasive Filter-Feeders, and the Effect of Parasites: Learning from Hypersaline Ecosystems
Source: PLoS One. 2016 Aug 25;11(8):e0161478. doi: 10.1371/journal.pone.0161478 (PMC4999065; doi:10.1371/journal.pone.0161478)
Supplement: S3 Table — Post hoc tests for the differences between taxa under different temperature treatments in the GLM of Table 1. See Table 1 for more details. Significant differences are shown in italics. (DOCX) [file pone.0161478.s003.docx]

**S3 Table**. **Post hoc tests for analysis from Table 1 under different temperature treatments**.

| \| TAXA \| \| --- \| | \| temperature \| \| --- \| | \| Ap15 \| \| --- \| | \| Ap24 \| \| --- \| | \| AfM15 \| \| --- \| | \| AfM24 \| \| --- \| | \| AfF15 \| \| --- \| | \| AfF24 \| \| --- \| |
| --- | --- | --- | --- | --- | --- | --- | --- | --- | --- | --- | --- | --- | --- | --- | --- |
| Ap | 15 |  | *0.0002* | *0.0005* | *< 0.0001* | *< 0.0001* | *< 0.0001* |
| Ap | 24 | *0.0002* |  | 0.7101 | *< 0.0001* | *< 0.0001* | *< 0.0001* |
| AfM | 15 | *0.0005* | 0.7101 |  | *< 0.0001* | *< 0.0001* | *< 0.0001* |
| AfM | 24 | *< 0.0001* | *< 0.0001* | *< 0.0001* |  | *< 0.0001* | *< 0.0001* |
| AfF | 15 | *< 0.0001* | *< 0.0001* | *< 0.0001* | *< 0.0001* |  | *< 0.0001* |
| AfF | 24 | *< 0.0001* | *< 0.0001* | *< 0.0001* | *< 0.0001* | *< 0.0001* |  |
