# Supplementary material for: Functional Role of Native and Invasive Filter-Feeders, and the Effect of Parasites: Learning from Hypersaline Ecosystems
Source: PLoS One. 2016 Aug 25;11(8):e0161478. doi: 10.1371/journal.pone.0161478 (PMC4999065; doi:10.1371/journal.pone.0161478)
Supplement: S4 Table — Results of GLM analysis on the number of cells consumed by A. parthenogenetica as a function of parasitic status (infected with F. liguloides FL or uninfected) at a salinity of 130 g/l. Parasitized status is aliased. Significant effects are shown in italics. (DOCX) [file pone.0161478.s004.docx]

**S4 Table**. **GLM on the number of cells consumed by *A. parthenogenetica* as a function of parasitic status (*F. liguloides* or uninfected)**.

|  | \| Estimate \| \| --- \| | \| SE \| \| --- \| | \| t _1, 95_ \| \| --- \| | \| p \| \| --- \| |
| --- | --- | --- | --- | --- | --- | --- | --- | --- |
| \| Intercept \| \| --- \| | -9637673 | 3380339 | -2.85 | *0.0052* |
| \| length (µm) \| \| --- \| | 1468 | 341 | 4.30 | *< 0.0001* |
| \| Uninfected status \| \| --- \| | 1542635 | 493146 | 3.13 | *0.0023* |
