# Supplementary material for: Functional Role of Native and Invasive Filter-Feeders, and the Effect of Parasites: Learning from Hypersaline Ecosystems
Source: PLoS One. 2016 Aug 25;11(8):e0161478. doi: 10.1371/journal.pone.0161478 (PMC4999065; doi:10.1371/journal.pone.0161478)
Supplement: S5 Table — Results of GLM analysis on the number of cells consumed by A. parthenogenetica as a function of parasitic status (infected with C. podicipina CP, A. tringae AT or uninfected) at a salinity of 130 g/l. Unparasitized status is aliased. Significant effects are shown in italics. (DOCX) [file pone.0161478.s005.docx]

**Table S5. GLM on the number of cells consumed by *A. parthenogenetica* as a function of parasitic status (*C. podicipina*, *A. tringae* or uninfected).**

|  | \| Level of Effect \| \| --- \| | \| Estimate \| \| --- \| | \| SE \| \| --- \| | \| F_2, 77_ \| \| --- \| | \| P \| \| --- \| |
| --- | --- | --- | --- | --- | --- | --- | --- | --- | --- | --- |
| \| Intercept \| \| --- \| |  | 486079 | 139409.3 | 12.16 | *0.0008* |
| \| length (µm) \| \| --- \| |  | -13 | 12.6 | 1.01 | 0.3177 |
| \| Parasitic status \| \| --- \| | CP | 97846 | 36867.2 | 6.57 | *0.0023* |
| \|  \| \| --- \| | AT | -106881 | 29649.5 |  |  |
