# Supplementary material for: Functional Role of Native and Invasive Filter-Feeders, and the Effect of Parasites: Learning from Hypersaline Ecosystems
Source: PLoS One. 2016 Aug 25;11(8):e0161478. doi: 10.1371/journal.pone.0161478 (PMC4999065; doi:10.1371/journal.pone.0161478)
Supplement: S6 Table — Post hoc tests for the differences between parasitic status (Confluaria podicipina CP, Anomotaenia tringae AT, non-infected NI) in the GLM of S4 Table. Significant differences are shown in italics. (DOCX) [file pone.0161478.s006.docx]

**S6 Table**. **Post hoc tests for analysis from S5 Table.**

| \| Parasitic status \| \| --- \| | \| CP \| \| --- \| | \| AT \| \| --- \| | \| NI \| \| --- \| |
| --- | --- | --- | --- | --- | --- | --- | --- |
| CP |  | *0.0054* | 0.6421 |
| AT | *0.0054* |  | *0.0246* |
| NI | 0.6421 | *0.0246* |  |
