# Supplementary material for: Functional Role of Native and Invasive Filter-Feeders, and the Effect of Parasites: Learning from Hypersaline Ecosystems
Source: PLoS One. 2016 Aug 25;11(8):e0161478. doi: 10.1371/journal.pone.0161478 (PMC4999065; doi:10.1371/journal.pone.0161478)
Supplement: S7 Table — Mean values (± SE) of salinity, chlorophyll-a concentration and Artemia density (A. franciscana and A. parthenogenetica) from April to December 2011 at La Tapa (invaded by A. franciscana) and Odiel (occupied by the native A. parthenogenetica). (DOCX) [file pone.0161478.s007.docx]

**S7 Table. Salinity, chlorophyll-a concentration and *Artemia* density at Odiel and Tapa.** Mean values (± SE) of salinity, chlorophyll-a concentration and Artemia density (*A. franciscana* and *A. parthenogenetica*) from April to December 2011 at La Tapa (invaded by *A. franciscana*) and Odiel (occupied by native *A. parthenogenetica*).

|  | *Tapa*  *________________________________* | | | *Odiel*  *_____________________________________* | | |
| --- | --- | --- | --- | --- | --- | --- |
| month | salinity | chlorophyll-a | *A. franciscana* | salinity | chlorophyll-a | *A. parthenogenetica* |
|  | (g/L) | (µg/L) | (ind/L) | (g/L) | (µg/L) | (ind/L) |
| April | 106 ± 51 | 4.8 ± 1.8 | 202 ± 167.3 | 85 ± 23 | 13.0 ± 7.5 | 894.8 ± 716.1 |
| May | 120 ± 10 | 4.6 ± 1.9 | 353 ± 277 |  |  |  |
| June | 172 ± 37 | 7.4 ± 1.6 | 197.8 ± 149.2 | 167 ± 37 | 21.4 ± 9.1 | 270.5 ± 156.2 |
| July | 155 ± 33 | 4.4 ± 1.7 | 138.5 ± 82 | 155 ± 35 | 7.2 ± 2.1 | 344.3 ± 307.7 |
| September | 151 ± 20 | 22.1 ± 4.5 | 147.8 ± 130.1 | 157 ± 37 | 14.3 ± 6.9 | 168 ± 168 |
| October | 223 ± 36 | 18.8 ± 4.4 | 0,0 | 192 ± 50 | 64.6 ± 19.4 | 174 ± 174 |
| November | 165 ± 32 | 42.5 ± 26.6 | 225 ± 194.6 | 175 ± 42 | 33.1 ± 13 | 84 ± 34.1 |
| December | 189 ± 22 | 9.4 ± 3.2 | 1313 ± 1030.5 | 186 ± 41 | 45.6 ± 9.4 | 36.8 ± 32.3 |
| Total | 160 ± 13 | 14.3 ± 4.7 | 322.1 ± 145.8 | 160 ± 13 | 28.5 ± 7.8 | 281.8 ± 109.5 |
